# Design of a Millimeter-Wave MIMO Antenna Array for 5G Communication Terminals

**DOI:** 10.3390/s22072768

**Published:** 2022-04-04

**Authors:** Jalal Khan, Sadiq Ullah, Usman Ali, Farooq Ahmad Tahir, Ildiko Peter, Ladislau Matekovits

**Affiliations:** 1 Department of Telecommunication Engineering, University of Engineering and Technology, Mardan 23200, Pakistan; jalal@uetmardan.edu.pk (J.K.); usman.ali@uetmardan.edu.pk (U.A.); 2 Research Institute for Microwave and Millimeter-Wave Studies (RIMMS), National University of Sciences and Technology (NUST), Islamabad 44000, Pakistan; farooq.tahir@rimms.nust.edu.pk; 3 Department of Industrial Engineering and Management, Faculty of Engineering and Information Technology, George Emil Palade University of Medicine, Pharmacy, Science, and Technology of Targu Mures, Str. N.Iorga nr. 1, 540139 Târgu Mureş, Romania; 4 Department of Electronics and Telecommunications, Politecnico di Torino, 10129 Turin, Italy; ladislau.matekovits@polito.it; 5 Istituto di Elettronica e di Ingegneria dell’Informazione e delle Telecomunicazioni, National Research Council of Italy, 10129 Turin, Italy; 6 Department of Measurements and Optical Electronics, Politehnica University Timisoara, 300006 Timisoara, Romania

**Keywords:** 5G, MIMO, antenna array, millimeter-wave, ECC, DG

## Abstract

This paper presents a design of multiple input multiple output (MIMO) antenna array for 5G millimeter-wave (mm-wave) communication systems. The proposed MIMO configuration consists of a two antenna arrays combination. Each antenna array consists of four elements which are arranged in an even manner, while two arrays are then assembled with a 90-degree shift with respect to each other. The substrate used is a 0.254 mm thick Rogers RT5880 with a dielectric constant of 2.2 and loss tangent of 0.0009, correspondingly. The proposed MIMO antenna array covers the 37 GHz frequency band, dedicated for 5G millimeter-wave communication applications. The proposed antenna element yields a gain of 6.84 dB, which is enhanced up to 12.8 dB by adopting a four elements array configuration. The proposed MIMO antenna array performance metrics, such as envelope correlation coefficient (ECC) and diversity gain (DG), are observed, which are found to be under the standard threshold. More than 85% of the radiation efficiency of the proposed MIMO antenna array is observed to be within the desired operating frequency band. All the proposed designs are simulated in computer simulation technology (CST) software. Furthermore, the measurements are carried out for the proposed MIMO antenna array, where a good agreement with simulated results is observed. Thus, the proposed design can be a potential candidate for 5G millimeter-wave communication systems.

## 1. Introduction

The wide interest for telecommunications and the latest telecommunication hardware in the ongoing years is obvious in the across the board expansion of cell phones making lives easy, operational, and intuitive by the internet. The CISCO Visual Networking Index (VNI), in 2017, predicted demands in terms of data handling for the year 2021. To establish the prerequisites in terms of cellular traffic, a concise portrayal of the present and determined traffic is as following: Globally, internet protocol (IP) video traffic has increased by 73 percent since 2016 and will increase to 82 percent by 2021. Traffic of virtual reality and augmented reality is predicted to increase 20 fold between 2016–2020; live internet video network will increase up to 15 fold between 2016–2020. Mobile data traffic, which is highly in demand, will increase quickly, to twice the current data traffic, by 2020 [1]. All these advancements clearly highlight the shifting to wireless communications. Keeping all these factors in mind, the demand for higher data rates, wide coverage, low latency, high reliability, and faster speed is increasing day by day, and present cellular networks are reaching the limits of their abilities [2,3,4,5]. Therefore, a shift towards the next generation of cellular technology is in demand.

5G millimeter-wave (mm-wave) technology has no clear definition yet, but it’s rather dealt with as the integration of multiple techniques, cases, and scenarios [6]. This technology will be using the unused millimeter waves spectrum, ranging from 30–300 GHz, in order to achieve higher data rates and bandwidths. Using higher frequencies for the next generation will result in free space path loss, which indicates that higher frequencies are greatly affected by atmospheric losses [7,8,9,10]. This means that higher frequencies are greatly suffering from atmospheric attenuation. In order to reduce path losses at higher frequencies, a technique of a high gain with a narrow beam radiation pattern produced by antenna is used [11,12,13,14,15]. However, using the array technique limits the channel capacity, which is one of the main aims of 5G; hence the array technique cannot utilize the multipath property, which can be helpful in enhancing the channel capacity [16,17,18]. Thus, the multiple antennas at the receiver and transmission side can be helpful in fulfilling capacity demand as well [19,20,21].

Here, we are also reporting some considerations on the materials that can be used in 5G antennas. The use of excellent materials is a very important task for the achievement of good functionalities in any communication tools, especially in a highly demanding one such as 5G communications. As mentioned above, with respect to the existing 4G network, 5G works at higher frequencies and spectrums, offering outstanding data aptitudes, unrestricted call capacities and information diffusion that is influenced using the most appropriate materials for the given request.

An innovative processing route is another task that has to be considered for the achievement of excellent results, with no signal losses and, at the same time, protecting signal reliability. Flexibility associated with wearable and implantable properties can amplify such evolution, with further benefits [22].

In communication, the choice and the growth in the antenna is a crucial consideration, and it is influenced by the atmosphere, the transmission power and/or the incidence range [23].

To date, for the development of a different type of antenna, with changing functionalities, various innovative materials have been employed. Graphene, making accessible high frequencies and bandwidths, advanced ceramics, guaranteeing the realization of ideal characteristics, glass and dielectric substrate materials, nonmetallic substrate materials, such as polymers, and metamaterials can be exploited for such an application, each of them presenting some advantages and, at the same time, some disadvantages. From the current literature date, some materials will be introduced below.

Graphene—a two dimensional single sheet material that is ordered in a hexagonal structure, guaranteeing an evident liberty to be packed into diverse forms—is a remarkable material for such an application. Additionally, it is light weight and shows good chemical stability, outstanding elasticity, and electrical and thermal conductivity which make possible the development of an antenna with reduced and thinner dimensions. Commonly, graphene based nano-antennas show the advantages of a smaller size than conventional microstrip antennas and demonstrate higher bandwidths and gains than metallic nanoantennas, but the methodologies employed for attaining a high quality product can limit large scale employment [24].

Carbon nanotubes (CNTs) are one dimensional materials and present rare electronic and electromagnetic characteristics such as high conductivity, defining very low resistive losses in the antenna. Compared to graphene, CNT based nanoantennas can operate at superior temperatures as a consequence of their higher thermal conductivity, which simplifies the dispersal of heat by the antenna surface [25].

Due to their low dielectric constant, low dissipation loss and good temperature stabilities, advanced ceramic materials can be exploited in several communication methods, making possible high frequency transmission. Piezoelectric ceramics, based on the lead zirconate titanate Pb(Zr1-xTixO3) system (PZT), in a perovskite (A2 + B4 + O3) structure, where A2+ or/and B4+ positions can be replaced with different dopants able to change the dielectric and piezoelectric characteristics, give rise to the development of innovative materials, obtained by a low cost processing route [26].

Metallic nanomaterials can be employed for the development of nanoantennas in the 5G network, generating remarkable characteristics, such as directivity gain, intensity expansions, decay speed improvement, and spectral modelling. Metamaterials, developed from different materials with specific properties, show singular electromagnetic properties, are capable of rising the behavior of nanoantennas, considerably reducing the size of the antenna and refining its performance, in terms of bandwidth, gain and by producing multiband frequencies.

Polymeric materials are well known as substrate materials for their high speed and stable signal transmission at microwave frequencies [27]. However, the Rogers family, as substrates with a low loss tangent, are also competent for mm-wave communication, as they can be quite helpful to contribute towards the achievement of the latest generation of promising features.

Greater interest in developing antenna designs for fifth generation technology has been shown by antenna researchers specifically at the 37–40 GHz frequency band [28,29,30,31,32,33]. Atmospheric losses for this frequency band are effectively minimum [34], which will be helpful for the future mm-wave based 5G communication to achieve its target of higher bandwidth provision and data rates more effectively. The researchers present an antenna operating in one of the 5G dedicated bands, i.e., 38 GHz, in [28]. The antenna presented is a single feed with no array or MIMO configuration, which could tackle the atmospheric attenuations challenge at mm-wave transmission and have a good capacity, which is one of the main aims of the latest communication technology. The overall peak gain of the reported antenna is, noticed below, 10 dB. An array antenna with a combination of four elements is presented in [29] which operates at the 38 GHz band. The gain achieved is more than 12 dB, which is enough for the mobile communication based on 5G but the single feed of the reported design limits its capacity. Likewise, the antenna reported in [30] covers the mm-wave band, i.e., 37–40 GHz, which is dedicated to 5G applications. The array configuration is adopted to increase the gain up to 12 dB but again, the single feed limits the capacity as no MIMO configuration is implemented. Furthermore, the size of the reported antenna array is 130 mm × 65 mm, which is quite large. Moreover, the antenna covering the 37–39 GHz band adopts an array structure for mm-wave applications to improve the gain [31]. The maximum gain achieved is 8.81 dB, while the antenna element reported in [32] adopts few metasurfaces to improve the gain but still the antenna element gain is not enhanced up to 12 dB and MIMO features are also lacking. The antenna presented in [33] can cover the band from 37.1–38.1 GHz with a size of 8 mm × 8 mm, but no gain enhancement or MIMO technique is implemented to tackle the atmosphere attenuations challenge and capacity limitations of single feed antennas.

Thus, this paper presents a simple MIMO antenna array designed at the 37 GHz band with four elements in the array structure using microstrip methods for 5G communication systems. The transmission line and quarter wavelength transformation techniques are used for impedance matching. The rest of the paper is organized as follows: Section 2, presents the design methodology of the proposed antenna. The results and discussion are presented in Section 3. While Section 4 concludes the paper.

## 2. Antenna Geometry

### 2.1. Single Element

**Figure 1:**
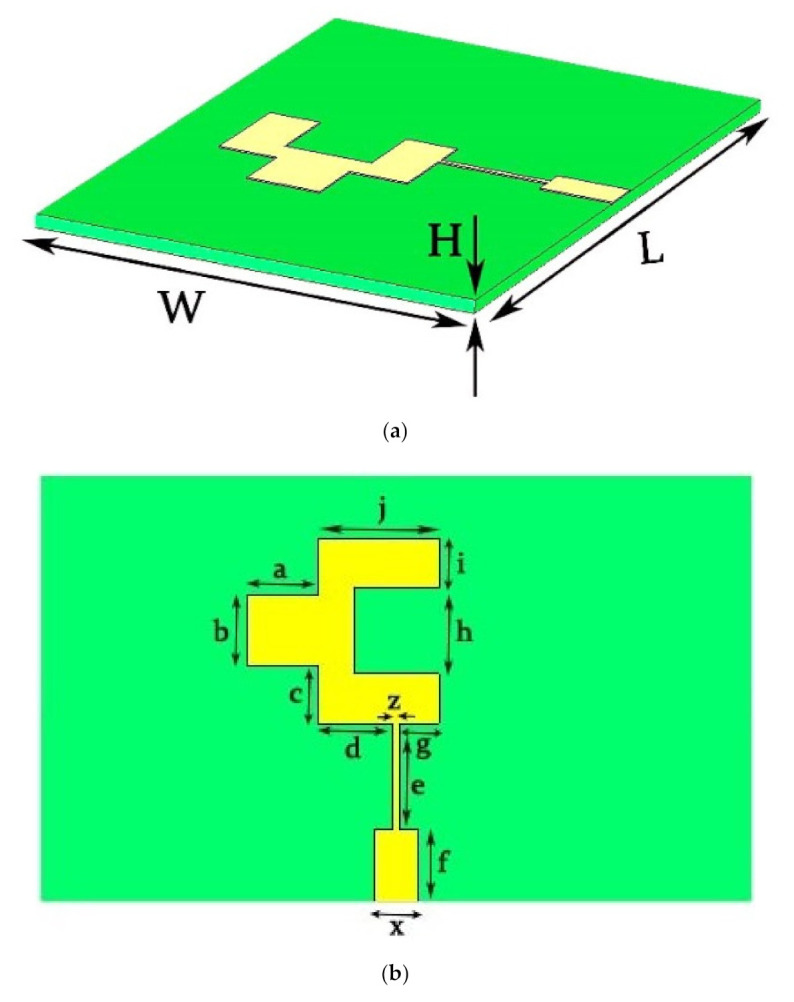

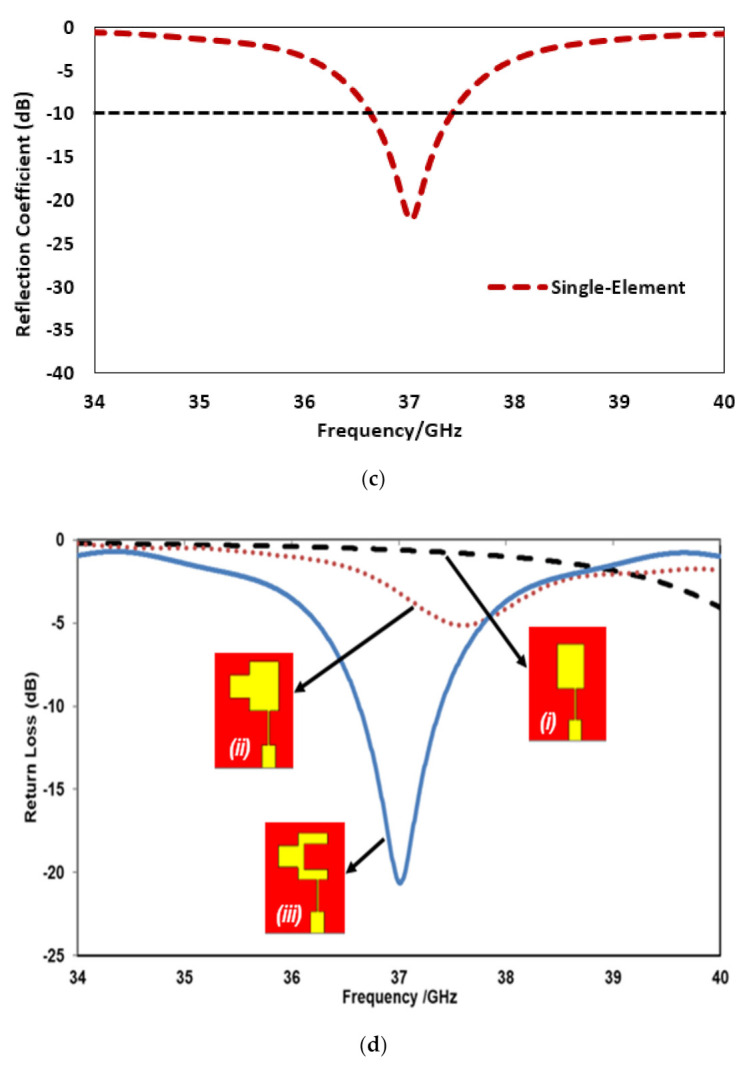
Geometry of the proposed antenna. (**a**) 3D view; (**b**) front view; (**c**) reflection coefficient; and (**d**) element evolution steps.

The antenna element designed for operation in the desired 37 GHz frequency band is demonstrated in Figure 1 and uses a 0.254 mm thick substrate from the Rogers family, i.e., RT-5880, with a loss tangent of 0.0009. However, the dielectric constant of the substrate used in the proposed design is 2.2. The full ground plane is supported at the back of the proposed antenna element. The length and width of the proposed antenna element is 10 × 6 (mm × mm), correspondingly. The purpose of the quarter wave transformation is to achieve impedance matching. The overall dimensions of the proposed antenna element is listed in Table 1 and are estimated using the famous transmission line theory for a rectangular patch antenna [35].

In Figure 1d, the designed element evolution steps are shown. Initially, in (i) the rectangular patch is designed, which gives a reflection coefficient above −10 dB and which is further improved in (ii) by introducing a strip to the left of the rectangular patch which improves the −10 dB bandwidth and then, finally, in (iii) the introduction of slot makes the proposed antenna element resonate with a satisfactory bandwidth.

### 2.2. Multiple Element Antenna Arrays

**Figure 2:**
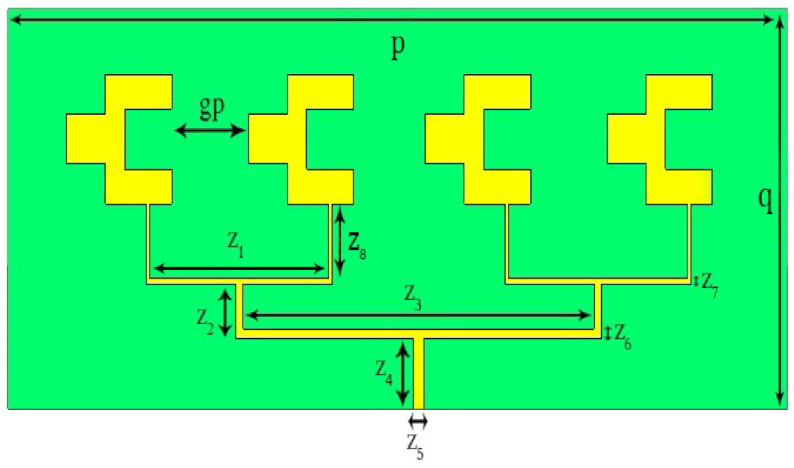
Structure of the four element array antenna.

The antenna element presented in Figure 1 yields a peak gain of 6.84 dB and it operates in the 37 GHz frequency band dedicated to 5G applications. The gain achieved is not enough to compete with the challenges that are possible at the mm-wave transmission. Thus, at the mobile level transmission, a minimum gain of 12 dB is usually preferred for satisfactory transmission. Therefore, there is a need to improve the gain of the single element; one of the simplest methods is the array technique [36], which utilizes multiple antenna elements in the form of an array to improve the gain, where the power is fed using a single feed. The geometry of the 1 × 4 array is presented in Figure 2. In the array structure, a power divider is involved which is optimized so as to make sure of the same power delivery to each antenna element, in order to have good radiation characteristics. The spacing between the elements is maintained to be 1.9 mm, which is 0.25 of the wavelengths at 37 GHz, in order to avoid coupling among the antenna elements, which is the main issue in the case of array structures that is tackled quite carefully in the proposed design of the 1 × 4 array by maintaining a suitable spacing of 1.9 mm. The parameters of the array antenna are outlined in Table 2.

The following relationship [37] is helpful to estimate the width of transmission lines in the proposed array structure, to ensure an equal power distribution:

(1) $$w_{z_{o}}=\left(\frac{377}{Z_{c}\sqrt{\varepsilon_{r}}}-2\right)\times h$$

**Figure 3:**
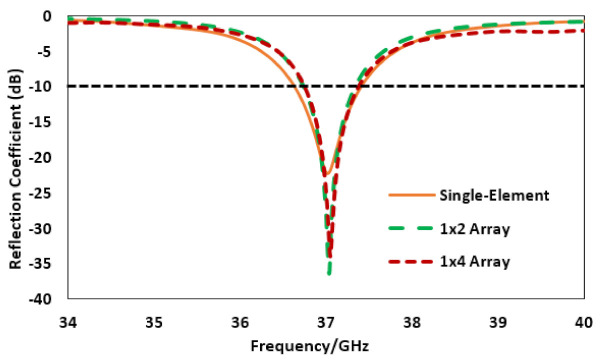
Reflection coefficient comparison for antenna different configurations.

The reflection coefficient comparison is analyzed in Figure 3 for the single element, 1 × 2 and 1 × 4 array structure. The level of the reflection coefficient is below −10 dB for all the proposed designs. The magnitude of the reflection coefficient noticed for the proposed antenna element is −22.24 dB at the 37 GHz band, while the reflection coefficient magnitude level for the proposed 1 × 2 and 1 × 4 array noticed is −36.24 dB, −33.23 dB, correspondingly, at the desired frequency band. The bandwidth observed based on the −10 dB criteria is 780 MHz, 653 MHz and 677 MHz for the single element, two and four element array antennas, correspondingly. The bandwidth in the case of the array structure is reduced a little bit due to the power divider, as minor losses incur while exciting each antenna element.

**Figure 4:**
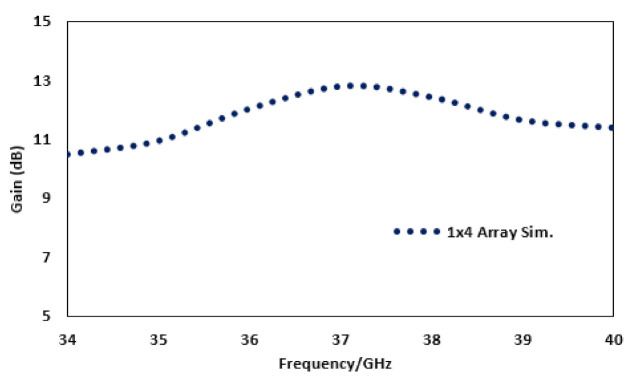
Gain of the four element antenna array.

The 1 × 4 array yields a peak gain of 12.8 dB, as shown in Figure 4, which is enough for the communication based on mm-wave transmission by 5G technology at the mobile communication level in order to ensure the signal integrity.

### 2.3. MIMO Configuration

**Figure 5:**
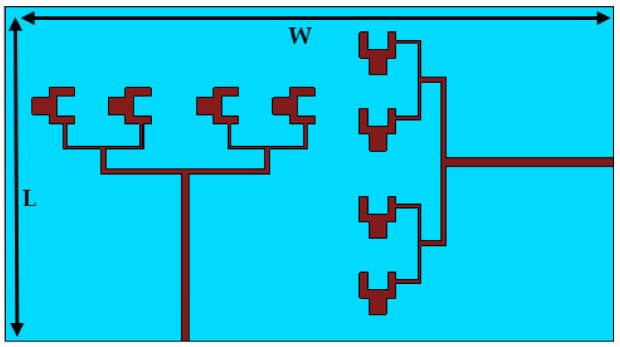
Corporate feed array MIMO configuration.

Figure 5 shows the extended design layout of the array design achieved utilizing the corporate feed technique to the MIMO configuration of two ports. The arrays are placed with a 90-degree shift with respect to each other in order to achieve pattern diversity and a good isolation among antenna arrays, which usually becomes a headache in cases of arrays that are extended to the MIMO. As in array structures, we already employ multiple elements to enhance the gain of the antenna but, due to their utilization of one port, channel capacity problems remain severe and, to address them efficiently, multiple ports are used. However, while that technique is little bit easy in the case of a single element, in the arrays, to control the coupling among the similar arrays is a challenging task. In the proposed MIMO structure, symmetry is maintained, and no further changes are made in the array structure above the position. The dimensions of the overall substrate used in MIMO configuration are L = 20 mm × W = 40 mm.

## 3. Results and Discussion

**Figure 6:**
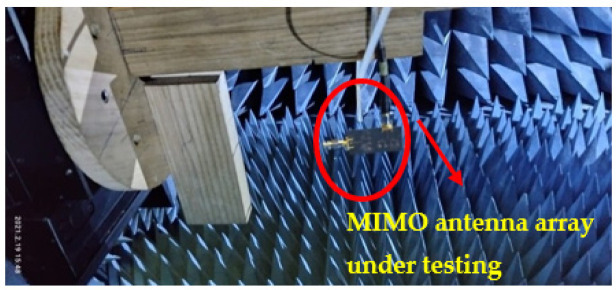
Proposed MIMO antenna array under testing.

In this section, the proposed MIMO antenna array is analyzed experimentally. The fabricated prototype under the test conditions is shown Figure 6. The antenna array MIMO configuration is excited by using a 50 Ω coaxial cable via a K-type connector, coupled with the transmission line. Various performance factors of the MIMO are also observed. A detailed comparison is also drawn between the simulated and measured results.

**Figure 7:**
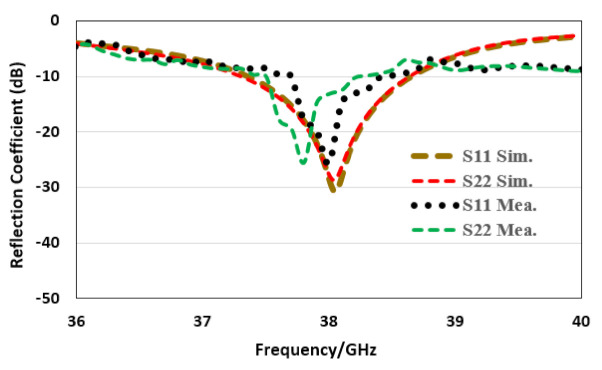
Corporate array MIMO configuration reflection coefficient.

The reflection coefficient for the proposed MIMO structure is presented in Figure 7. For port-1, the antenna resonates with the central frequency of 38.04 GHz, giving a bandwidth from 38.35 to 38.66 GHz. Whereas the magnitude achieved in −31 dB. Similarly, for port-2, the antenna resonates with the central frequency of 38.03 GHz with a reflection coefficient magnitude of −28 dB, while the bandwidth is from 37.29 to 38.64 GHz below the 10 dB.

**Figure 8:**
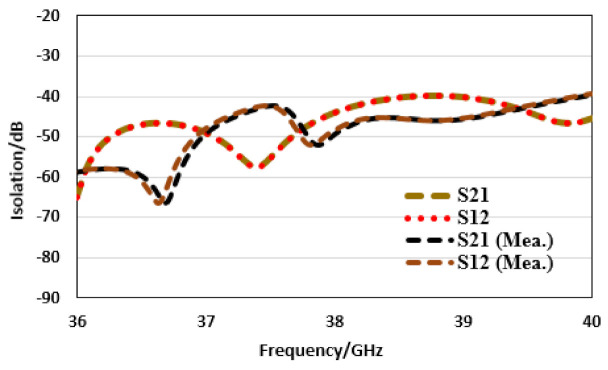
Corporate array MIMO isolation analysis.

The isolation, i.e., mutual coupling analysis, becomes especially important in the case of array MIMO configuration, when more than one antenna array are placed within close proximity of each other. That can cause the mutual coupling affects to be severe and to degrade the performance of the antenna in terms of efficiency, gain, etc. That is why the isolation for the proposed MIMO antenna is presented and analyzed in Figure 8, for both the ports with respect to each other. The isolation is observed to be greater than 40 dB within the operating bandwidth, which clearly indicates that the isolation among the arrays employed is quite minimum. Overall, the measured results follow the simulation quite satisfactorily, while the little discrepancy is due to connector or fabrication losses.

**Figure 9:**
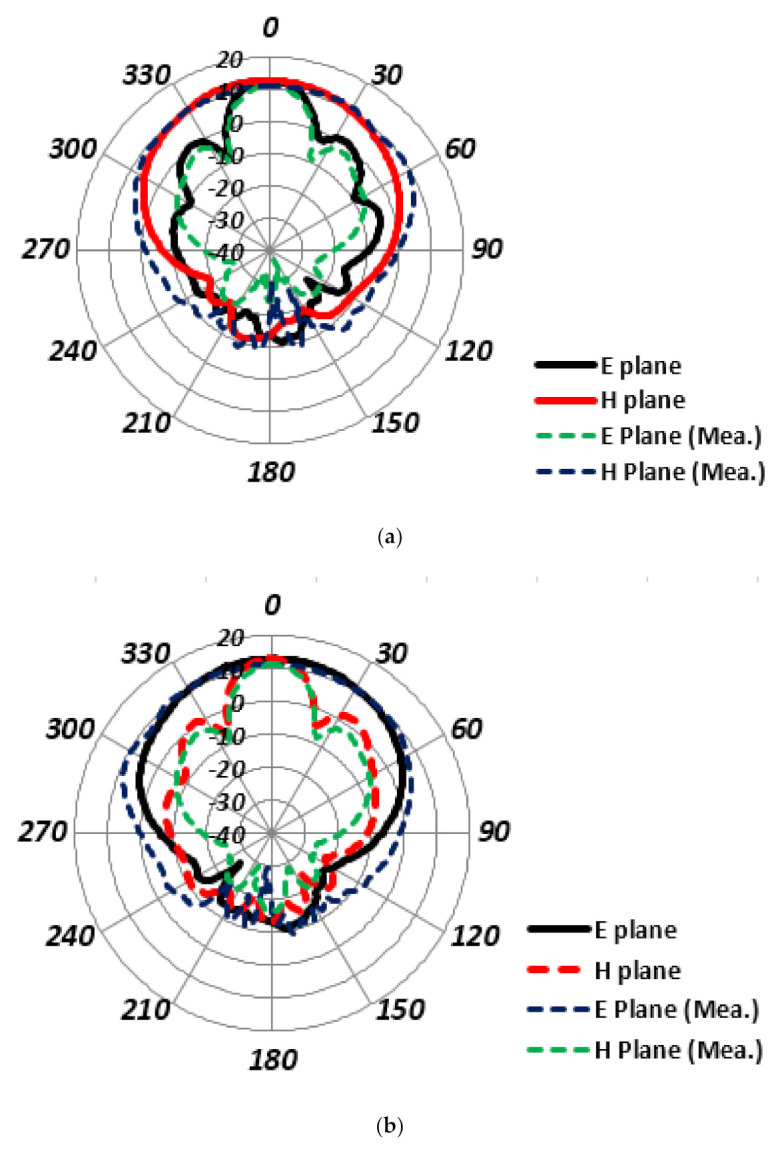
Corporate array MIMO configuration gain pattern for: (**a**) port-1, (**b**) port-2.

The gain patterns are analyzed and presented in Figure 9a, in the 0- and 90-degree planes for the port-1. Initially, in the 90-degree plane, the antenna array main lobe direction is located at the 353.0 degrees. While a very good side lobe level of −24.3 dB is achieved with a 3 dB beamwidth of 68.2 degrees. The radiation pattern is quite directive, with the minimum back lobes and side lobes. The 0-degree plane analysis shows us that the main lobe is directed towards the 359-degree angle and the side lobe level is −11.7 dB. Furthermore, the 3 dB beamwidth is 22 degrees. In this plane, the radiated beam also sees to be highly directive, with a low level of back lobes.

Similarly, the radiation pattern analysis in the case of port-2 is presented in Figure 9b for the two main planes, E and H. The main lobe direction for the 0-degree plane is along the 4.0-degree angle and the side lobe level is −23.8 dB. Whereas the angular width is 69.3 degrees in this plane. Likewise, the direction of the main lobe is along 359.0-degree angle in the 90-degree plane, with an angular width of 20.6 degrees. The side lobe level is −10.7 degree in this plane. The measured radiation patterns follow the simulated ones, which validate the simulated results. The radiation efficiency of the proposed MIMO antenna array is observed to be more than 85% within the desired operating frequency band.

**Figure 10:**
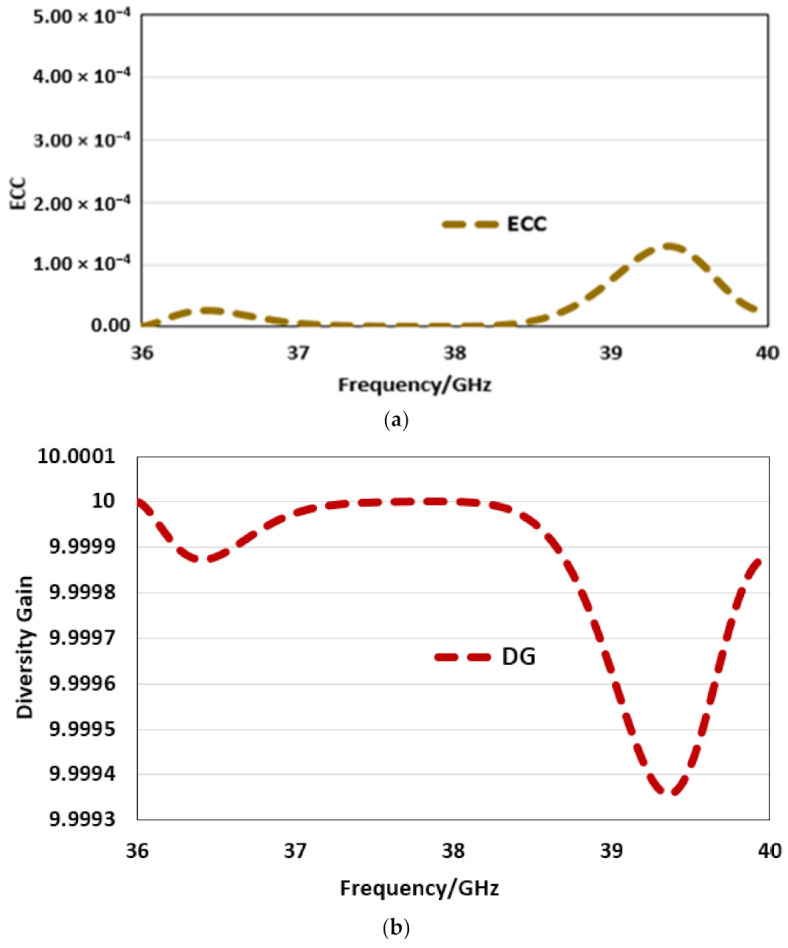

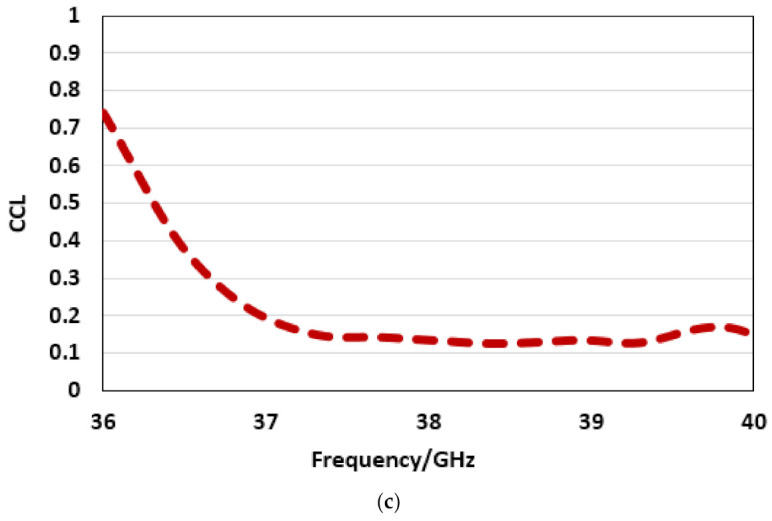
Corporate array MIMO configuration: (**a**) ECC, (**b**) DG, (**c**) CCL. 1.00 × 10^−4^.

The envelope correlation coefficient stands for ECC is another parameter [38] that describes the level of correlation among the multiple antennas that come in proximity of each other. For the proposed MIMO configuration of the two port antenna array, the ECC value achieved is below 0.00014 for the entire operating bandwidth, which satisfies the standard value criteria of less than 0.5 and depicts that isolation among the antenna arrays is minimum, as shown in Figure 10a.

Another performance metric of the MIMO is diversity gain (DG) [39], which shows that, if the standard value of 10 dB is met or is close to this, the reduction in the transmitted power will have no major effect on the quality of the transmission or MIMO performance. As seen in Figure 10b, the DG value is close to 10 for the entire operating bandwidth, which satisfies the standard criteria. The channel capacity loss (CCL), being another performance parameter [40], is shown in Figure 10c and also depicts good performance. All the results discussed above show that the MIMO based corporate feed arrays are very suitable for future 5G millimeter wave communication. The proposed antenna can be optimized to give dual band operation in the standard 5G bands by using either the approach of double-layer gridded patches [41] or the use of overlapped apertures [42].

## 4. Conclusions

In this work, a design of a multiple input multiple output (MIMO) antenna array for 5G millimeter-wave (mm-wave) communication systems is presented. The proposed MIMO configuration consists of a two antenna arrays combination. Each antenna array consists of four elements, which are arranged in an even manner, while two arrays are then assembled with a 90-degree shift with respect to each other. The proposed MIMO antenna array covers the 37 GHz frequency band, dedicated to 5G mm-wave communication applications. The proposed antenna element yields a gain of 6.84 dB, which is enhanced up to 12.8 dB by adopting the four elements array configuration. The proposed MIMO antenna array performance metrics, such as envelope correlation coefficient (ECC) and diversity gain (DG), are observed and are found to be under the standard threshold. The radiation efficiency of the proposed MIMO antenna array is observed to be more than 85% within the desired operating frequency band. Furthermore, the measurements are carried out for the proposed MIMO antenna array, where a good agreement with simulated results is observed. Thus, the proposed design can be a potential candidate for 5G mm-wave communication systems.

## Figures and Tables

**Table 1:** Summary of the dimensions of the proposed design.

Parameter	L	W	H	a	b	c	d	e
Value (mm)	10	6	0.254	1	1	0.8	1.05	1.5
Parameter	f	G	i	j	k	s	w	z
Value (mm)	1	0.55	0.7	1.7	1.22	1.21	0.62	0.1

**Table 2:** Design parameters of four element array antenna.

Parameter	p	q	z_1_	z_2_	z_3_	z_8_
Value (mm)	20	8	4.56	1.1	90	1.6
Parameter	z_4_	z_5_	z_6_	z_7_	gp	-
Value (mm)	1.4	0.27	0.2	0.1	1.9	

## Data Availability

Data is contained within the article.

## References

[1] Storck C.R., Duarte-Figueiredo F. (2020). A survey of 5G technology evolution, standards, and infrastructure associated with vehicle-to-everything communications by internet of vehicles. IEEE Access.

[2] Barreto A.N., Faria B., Almeida E., Rodriguez I., Lauridsen M., Amorim R., Vieira R. (2016). 5G—Wireless Communications for 2020. J. Commun. Inf. Syst..

[3] Patnaik P., Sarkar D., Saha C. A multi-band 5G antenna for Smart phones operating at Sub-6 GHz frequencies. Proceedings of the IEEE International Symposium on Antennas & Propagation (APSYM).

[4] Nam H.J., Lim S., Yoon Y.J., Kim H. Tunable triple-band antenna for Sub-6 GHz 5G mobile phone. Proceedings of the IEEE International Symposium on Antennas and Propagation and North American Radio Science Meeting.

[5] Jiang J., Li Y. A wideband kanji patch antenna for 5G Sub-6-GHz applications. Proceedings of the IEEE 13th UK-Europe-China Workshop on Millimetre-Waves and Terahertz Technologies (UCMMT).

[6] Sehrai D.A., Abdullah M., Altaf A., Kiani S.H., Muhammad F., Tufail M., Irfan M., Glowacz A., Rahman S. (2020). A novel high gain wideband MIMO antenna for 5 G millimeter wave applications. Electronics.

[7] Mao C., Gao S., Wang Y. (2017). Broadband high-gain beam-scanning antenna array for millimeter-wave applications. IEEE Trans. Antennas Propag..

[8] Zhu J., Chu C., Deng L., Zhang C., Yang Y., Li S. (2018). mm-wave high gain cavity-backed aperture-coupled patch antenna array. IEEE Access.

[9] Raheel K., Altaf A., Waheed A., Kiani S.H., Sehrai D.A., Tubbal F., Raad R. (2021). E-shaped H-slotted dual band mmWave antenna for 5G technology. Electronics.

[10] Di Paola C., Zhao K., Zhang S., Pedersen G.F. (2019). SIW multibeam antenna array at 30 GHz for 5G mobile devices. IEEE Access.

[11] Yang S.J., Pan Y.M., Shi L., Zhang X.Y. (2020). Millimeter-wave dual-polarized filtering antenna for 5G application. IEEE Trans. Antennas Propag..

[12] Ali M., Muñoz L.E.G., Carpintero G., Nellen S., Globisch B. Millimetre-wave photonic emitter integrating a PIN-PD and planar high gain antenna. Proceedings of the IEEE Third International Workshop on Mobile Terahertz Systems (IWMTS).

[13] Johnson M., Dascurcu J., Zhan K., Galioglu A., Adepu N., Jain S., Krishnaswamy H., Natarajan A.S. (2020). Code-domain multiplexing for shared IF/LO interfaces in millimeter-wave MIMO arrays. IEEE J. Solid-State Circuits.

[14] Zhang J., Huang Y., Wang J., Schober R., Yang L. (2020). Power-efficient beam designs for millimeter wave communication systems. IEEE Trans. Wirel. Commun..

[15] Xu H., Yu J., Zhu S. (2019). Ping-pong optimization of user selection and beam allocation for millimeter wave communications. IEEE Access.

[16] Zhao X., Yeo S.P., Ong L.O. (2017). Decoupling of inverted-F antennas with high-order modes of ground plane for 5G mobile MIMO platform. IEEE Trans. Antennas Propag..

[17] Li M.Y., Xu Z.Q., Ban Y.L., Sim C.Y.D., Yu Z.F. (2017). Eight-port orthogonally dual-polarized MIMO antenna using loop structures for 5G smartphone. IET Microw. Antennas Propag..

[18] Li M.Y., Ban Y.L., Xu Z.Q., Guo J., Yu Z.F. (2017). Tri-polarized 12-Antenna MIMO array for future 5G smartphone applications. IEEE Access.

[19] Jiang W., Cui Y., Liu B., Hu W., Xi Y. (2019). A dual-band MIMO antenna with enhanced isolation for 5G smartphone applications. IEEE Access.

[20] Li H., Tsiaras A., Lau B.K. (2017). Analysis and estimation of MIMO-SAR for multi-antenna mobile handsets. IEEE Trans. Antennas Propag..

[21] Li M.Y., Li C., Ban Y.L., Kang K. Multiple antennas for future 4G/5G smartphone applications. Proceedings of the IEEE MTT-S International Microwave Workshop Series on Advanced Materials and Processes for RF and THz Applications (IMWS-AMP).

[22] Corchia L., Monti G., Tarricone L. (2019). Wearable antennas: Nontextile versus fully textile solutions. IEEE Antennas Propag..

[23] Wang M., Ma H.F., Zhang H.C., Tang W.X., Zhang X.R., Cui T.J. (2018). Frequency-fixed beamscanning leaky-wave antenna using electronically controllable corrugated microstrip line. IEEE Trans. Antennas Propag..

[24] Wang J., Lu W., Liu Z., Zhang A., Chen H. (2019). Graphene-based microwave antennas with reconfigurable pattern. IEEE Trans. Antennas Propag..

[25] Tang L., Zhang J., Tang Y., Kong J., Liu T., Gu J. (2021). Polymer matrix wave-transparent composites: A review. J. Mater. Sci. Technol..

[26] Dumitru A.I., Velciu G., Pintea J., Patroi D., Marinescu V., Clicinschi F., Matekovits L., Peter I. (2020). Investigations on the doping effects on the properties of piezoelectric ceramics. Adv. Mater. Res..

[27] Park T.H., Kim S.M., Oh M.C. (2020). Polymeric tunable wavelength filter with two-stage cascaded tilted Bragg gratings. Opt. Express.

[28] Sharaf M.H., Zaki A.I., Hamad R.K., Omar M.M.M. (2020). A novel dual-band (38/60 GHz) patch antenna for 5G mobile handsets. Sensors.

[29] Khan J., Sehrai D.A., Ali U. (2019). Design of dual band 5G antenna array with SAR analysis for future mobile handsets. J. Electr. Eng. Technol..

[30] Peng M., Zhao A. High performance 5G millimeter-wave antenna array for 37–40 GHz mobile application. Proceedings of the IEEE International Workshop on Antenna Technology (iWAT).

[31] Park J., Choi D., Hong W. 37–39 GHz vertically-polarized end-fire 5G antenna array featuring electrically small profile. Proceedings of the IEEE International Symposium on Antennas and Propagation & USNC/URSI National Radio Science Meeting, Marina Bay Sands.

[32] Khan J., Sehrai D.A., Khan M.A., Khan H.A., Ahmad S., Ahmad S., Ali A., Arif A., Memon A.A., Khan S. (2019). Design and performance comparison of rotated Y-shaped antenna using different metamaterial surfaces for 5G mobile devices. Comput. Mater. Contin..

[33] Nosrati M., Tavassolian N. A single feed dual-band, linearly/circularly polarized cross-slot millimeter-wave antenna for future 5G networks. Proceedings of the IEEE International Symposium on Antennas and Propagation & USNC/URSI National Radio Science Meeting.

[34] Sehrai D.A., Asif M., Shoaib N., Ibrar M., Jan S., Alibakhshikenari M., Lalbakhsh A., Limiti E. (2021). Compact Quad-Element High-Isolation Wideband MIMO Antenna for mm-Wave Applications. Electronics.

[35] Ballanis C.A. (2016). Antenna Theory Analysis and Design.

[36] Ojaroudiparchin N., Shen M., Pedersen G.F. Beam-steerable microstrip-fed bow-tie antenna array for fifth generation cellular communications. Proceedings of the IEEE 10th European Conference on Antennas and Propagation (EuCAP).

[37] Kamal M.M., Yang S., Kiani S.H., Sehrai D.A., Alibakhshikenari M., Abdullah M., Falcone F., Limiti E., Munir M. (2021). A Novel Hook-Shaped Antenna Operating at 28 GHz for Future 5G mmwave Applications. Electronics.

[38] Ullah U., Al-Hasan M., Koziel S., Mabrouk I.B. (2021). Series-slot-fed circularly polarized multiple-input–multiple-output antenna array enabling circular polarization diversity for 5G 28 GHz indoor applications. IEEE Trans. Antennas Propag..

[39] Hussain N., Jeong M., Abbas A., Kim N. (2020). Metasurface-based single-layer wideband circularly polarized MIMO antenna for 5G millimeter-wave systems. IEEE Access.

[40] Dicandia F.A., Genovesi S., Monorchio A. (2017). Analysis of the performance enhancement of MIMO systems employing circular polarization. IEEE Trans. Antennas Propag..

[41] Sun W., Li Y., Chang L., Li H., Qin X., Wang H. (2021). Dual-band dual-polarized microstrip antenna array using double-layer gridded patches for 5G millimeter-wave applications. IEEE Trans. Antennas Propag..

[42] Li H., Li Y., Chang L., Sun W., Qin X., Wang H. (2020). A wideband dual-polarized endfire antenna array with overlapped apertures and small clearance for 5G millimeter-wave applications. IEEE Trans. Antennas Propag..

